# P-1728. Let's Settle the "Score": Implementation of a Prioritization Tool into an Antimicrobial Stewardship Prospective Audit and Feedback Program

**DOI:** 10.1093/ofid/ofae631.1892

**Published:** 2025-01-29

**Authors:** Alexander R Cain, Kimberly C Claeys, Megan Dunning, Emily L Heil, Mandee Noval, Jonathan S Lapin, Sara Lee, Ravi Tripathi, Jacqueline T Bork

**Affiliations:** University of Maryland Medical Center, Baltimore, Maryland; University of Maryland Baltimore, Baltimore, Maryland; University of Maryland Medical Center, Baltimore, Maryland; University of Maryland School of Pharmacy, Baltimore, MD; University of Maryland School of Pharmacy, Baltimore, MD; University of Maryland Medical Center, Baltimore, Maryland; University of Maryland Medical Center, Baltimore, Maryland; University of Maryland School of Medicine, Baltimore, Maryland; University of Maryland School of Medicine, Baltimore, Maryland

## Abstract

**Background:**

Prospective audit and feedback (PAF) remains the backbone of antimicrobial stewardship (AS) action. However, PAF is time consuming and does not always result in intervention. A prioritization scoring tool is a proposed solution to improve AS efficiency. We sought to assess the impact of a scoring tool (‘score’) on the efficiency of our AS program.

**Figure 1.**
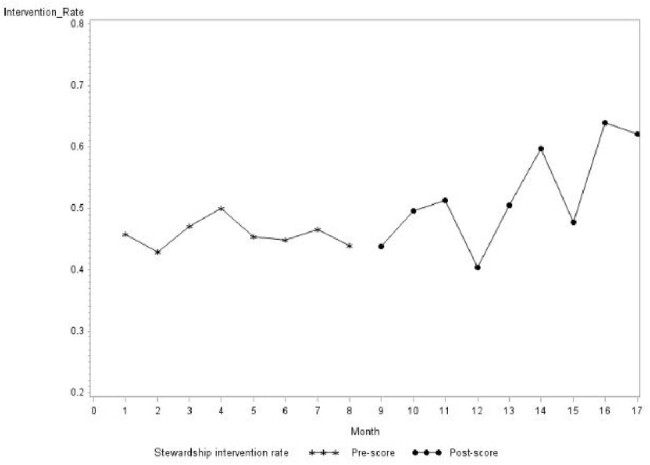
Interrupted Time Series Analysis of Intervention Rate Pre-Post Score Implementation

**Methods:**

A pre-post evaluation of AS PAF at an academic medical center was performed. The pre-score period (November 2022 – June 2023) consisted of standard PAF (largely 48-hour review). The post-score period (July 2023 – March 2024) included standard PAF in addition to the score. The scoring tool is embedded within the AS surveillance system in the electronic medical record and is used to readily identify patients with opportunities for antimicrobial optimization. It consists of pre-defined “points” for each programmed alert (e.g. bug-drug mismatch=10) which are reported in real-time as a prioritized list (higher score = higher priority). An interrupted time series was conducted to compare the intervention rate pre- and post-score implementation. A subgroup analysis was conducted on the post-score period comparing the reviews triggered by the score versus the 48-hour review. A univariate logistic regression was carried out to determine the association of the score with AS intervention.

**Table 1.**
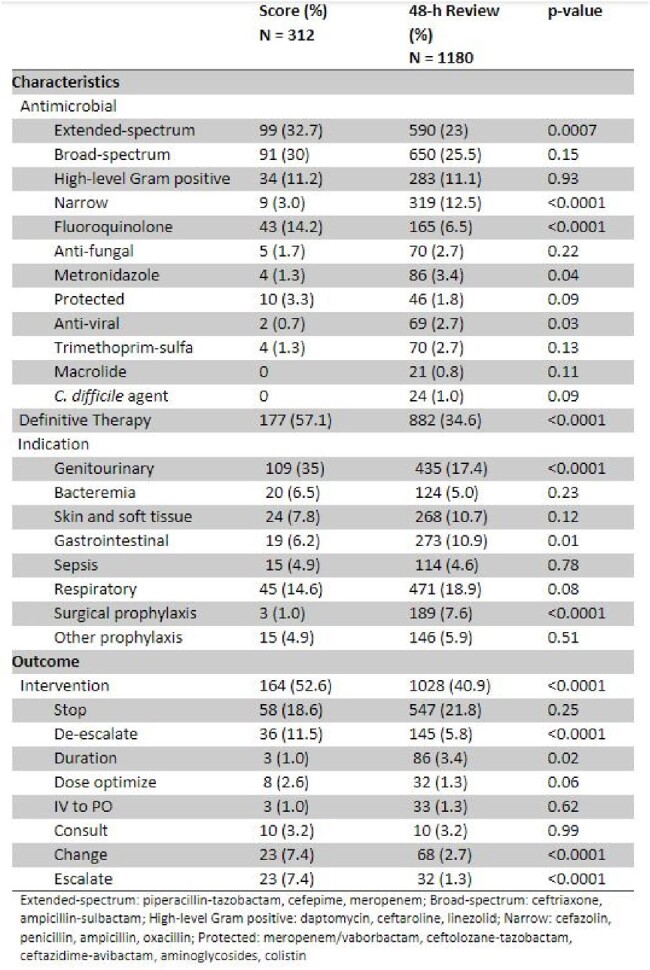
Comparison of Score Interventions vs 48-hour Review during the Post-Period

**Results:**

There were 5801 AS reviews in total, 2927 pre-score and 2874 post-score (92 missing outcome data). The mean intervention rate pre- and post-score was 46.2% and 52.3%, respectively (p< 0.001), with a +2.2% difference per month (p=0.036) as illustrated by Figure 1. The change was gradual without any significant, immediate level change. The score subgroup consisted of 312 reviews and were compared to 1180 48-hour reviews during the same period. Table 1 compares the two groups demonstrating overall high intervention rate in the score group (52.6% vs 40.9%, p < 0.0001); however, notable characteristics were different. An AS intervention was 1.6 times more likely when using the score approach (OR 1.6, 95% CI 1.3 – 2.0).

**Conclusion:**

Implementation of an antimicrobial scoring tool may improve PAF efficiency by better predicting patients for antimicrobial optimization than standard 48-hour review. The scoring tool can be an impactful method when performing PAF.

**Disclosures:**

**Kimberly C. Claeys, PharmD, PhD**, bioMérieux: Advisor/Consultant|bioMérieux: Honoraria

